# Clinical diagnostic value of American College of Radiology thyroid imaging report and data system in different kinds of thyroid nodules

**DOI:** 10.1186/s12902-022-01053-z

**Published:** 2022-05-31

**Authors:** Ziwei Zhang, Ning Lin

**Affiliations:** Ultrasonography Department, Fujian Provincial Hospital, 134 Fuzhou East Street, Fuzhou, 350001 China

**Keywords:** Diagnostic value, MTC, PTC, ACR score, ACR TI-RADS

## Abstract

**Background:**

To evaluate the diagnostic value of American College of Radiology (ACR) score and ACR Thyroid Imaging Report and Data System (TI-RADS) for benign nodules, medullary thyroid carcinoma (MTC) and papillary thyroid carcinoma (PTC) through comparing with Kwak TI-RADS.

**Methods:**

Five hundred nine patients diagnosed with PTC, MTC or benign thyroid nodules were included and classified into the benign thyroid nodules group (*n* = 264), the PTC group (*n* = 189) and the MTC group (*n* = 56). The area under the curve (AUC) values were analyzed and the receiver operator characteristic (ROC) curves were drawn to compare the diagnostic efficiencies of ACR score, ACR TI-RADS and KWAK TI-RADS on benign thyroid nodules, MTC and PTC.

**Results:**

The AUC values of ACR score, ACR TI-RADS and Kwak TI-RADS for distinguishing malignant nodules from benign nodules were 0.914 (95%CI: 0.886–0.937), 0.871 (95%CI: 0.839–0.899) and 0.885 (95%CI: 0.854–0.911), respectively. In distinguishing of patients with MTC from PTC, the AUC values of ACR score, ACR TI-RADS and Kwak TI-RADS were 0.650 (95%CI: 0.565–0.734), 0.596 (95%CI: 0.527–0.664), and 0.613 (95%CI: 0.545–0.681), respectively. The AUC values of ACR score, ACR TI-RADS and Kwak TI-RADS for the discrimination of patients with MTC, PTC or benign nodules from patients without MTC, PTC or benign nodules were 0.899 (95%CI: 0.882–0.915), 0.865 (95%CI: 0.846–0.885), and 0.873 (95%CI: 0.854–0.893), respectively.

**Conclusion:**

The ACR score performed the best, followed ex aequo by the ACR and Kwak TI-RADS in discriminating patients with malignant nodules from benign nodules and patients with MTC from PTC.

**Supplementary Information:**

The online version contains supplementary material available at 10.1186/s12902-022-01053-z.

## Background

Thyroid nodule is a kind of disease with scattered lesions caused by local abnormal growth of thyroid cells with a high incidence in the general population [1]. The detection rate of thyroid nodule is 3%-7% by palpation, and can be as high as 20%-76% by high-resolution ultrasound [2]. Approximately 1.6%-12% thyroid nodules are reported to be malignant, which refers to thyroid cancer [3]. Due to the increasing application of imaging diagnostic technology, the incidence of thyroid cancer continues to rise worldwide [4]. Papillary thyroid carcinoma (PTC), arising from the thyroid follicular cells, is the most common primary thyroid malignancy, accounting for more than 80% of thyroid cancers, and PTC patients generally have a good prognosis [5]. Medullary thyroid carcinoma (MTC), arising from the parafollicular C cells, and represents 3%-5% of all thyroid cancer cases, occurs in familial and sporadic forms, and about 13.4% deaths in thyroid cancers was caused by MTC [6]. MTC has diverse features, which can be similar to arbitrary thyroid malignancy and many MTC cases are easily missed or delayed diagnosis due to lacking malignant ultrasonography features [7]. Therefore, to accurately identify patients with MTC is of great significance for the improvement of disease treatments and patients’ prognosis.

Nowadays, regular medical examination in general population are more and more popular in China, and more patients with thyroid nodules were identified through palpation [8]. For newly diagnosed patients, to discriminate benign or malignant nodules was necessary. Ultrasonography is an imaging modality for measuring thyroid nodules and sonographic findings including location, composition, echogenicity, margins, calcifications, shape, vascularity and size are evaluated to stratify malignancy risk based on the Thyroid Imaging Report and Data System (TI-RADS) [9]. TI-RADS helps improve the diagnostic effectiveness of ultrasound and reduce unnecessary preoperative fine-needle aspiration (FNA) biopsies [9]. To date, several classification systems have been proposed to achieve standardized evaluation of clinical ultrasound, including Kwak TI-RADS proposed by Kwak et al. [10], the classification of thyroid diagnosis and treatment guidelines proposed by the American Thyroid Society (ATA) and recently updated American College of Radiology (ACR) TI-RADS [11]. Currently, TI-RADS is mostly applied for distinguishing and diagnosing of benign and malignant thyroid nodules [12]. In a previous study, the diagnostic efficacies of Kwak TI-RADS and ATA guidelines have been compared in the diagnosis of PTC and MTC [13]. ACR TI-RADS has improved the diagnostic performance of ultrasonic prediction of thyroid malignant nodule with high accuracy and high repeatability [14]. Although calcitonin (CT) is the specific serum marker of MTC, it was preoperative performed in patients with suspected nodules (classified as TIRADS 4 or large nodules with a diameter of more than 3 cm). For newly diagnosed patients with thyroid nodules, the diagnostic ability of ACR TI-RADS for MTC still needs investigation.

In this study, the pathologically confirmed benign and malignant thyroid nodules patients were included to compare the ultrasonic characteristics of different types of thyroid nodules, and evaluate the diagnostic value of ACR TI-RADS for MTC and PTC through comparing with Kwak TI-RADS. The findings of our study might provide a reference for the application of ACR TI-RADS in the diagnosis of MTC patients in clinic.

## Methods

### Study population

In the present study, the clinical data, laboratory inspection data and supersonic inspection data of 565 patients diagnosed with PTC, MTC or benign thyroid nodules were collected in Fujian Provincial Hospital. After excluding patients with a history of other malignancies or receiving adjuvant therapy such as chemotherapy before surgery, 509 patients were finally included. All patients were classified into the benign nodules group (*n* = 264), the PTC group (*n* = 189) and the MTC group (*n* = 56). The study was approved by the Ethics Committee of Fujian Provincial Hospital (K2020-01–027). All methods were performed in accordance with the relevant guidelines and regulations.

### Ultrasound and pathological report analysis

High resolution ultrasound examinations of the thyroid were performed by use of a HDI 3000 scanner (Advanced Technology Laboratories, Philips Medical Systems, Bothell, WA) and a HDI 5000 scanner (Philips Medical Systems) with a 5–10 MHz linear array transducer. Radiologists reported the ultrasound records based on the TI-RADS classification [15].

### Data collection

The clinical data, laboratory inspection data and supersonic inspection data of 565 patients were collected including age (years), gender, ACR TI-RADS score, ACR TI-RADS classification system (TR1, TR2, TR3, TR4, and TR5), Kwak TI-RADS [type 2, type 3, type 4 (type 4a, type 4b and type 4c), and type 5], carcinoma embryonic antigen (CEA; ng/mL; positive, negative or unknown), calcitonin (CT; pg/mL; positive, negative or unknown), thyroid stimulating hormone (TSH; μIU/L; positive, negative or unknown), thyrotropin receptor antibody (TRAb; IU/L; positive, negative or unknown), thyroid peroxidase antibody (TPOAb; IU/mL; positive, negative or unknown), thyroglobulin antibodies (TGAb; IU/mL; positive, negative or unknown), maximum diameter of nodule (cm), composition (cystic, spongy, solid cystic and solid), echogenicity (anechoic, hyperechoic, hypoechoic and markedly hypoechoic), shape (A/T < 1 or A/T ≥ 1), margins (smooth or unclear, irregular or lobulated and extrathyroid extension), calcification (none, coarse calcifications, rim calcifications and microcalcifications), and metastatic cervical lymph nodes or not.

### Evaluation of the laboratory data

The level of TSH was determined via ultra-sensitive chemiluminescence immunoassay (Roche Diagnostics, Mannheim, Germany) (normal ranges 0.27–4.2mIU/L). CT, CEA, TPOAb, TGAb, and TRAb were detected through immunoassay analysis on a fully automated analyzer Cobas e601 (Roche Diagnostics, Rotkreuz, Switzerland) using electrochemiluminescence (ECL) technology. The Elecsys® Calcitonin assay was standardized against the International Reference Preparation (IRP) World Health Organization 89/620 international standard. The range of CT ≥ 9.52 pg/mL in males or ≥ 6.4 pg/mL in females were considered positive. The range of CEA ≥ 5 ng/mL was regarded as positive. The normal range was considered if TPOAb was 0–34 IU/mL, TRAb was 0–1.75 U/L and TGAb was 0.1–115 IU/mL. All assays were performed in line with the manufacturers’ instructions.

### Definitions of ultrasound characteristics

The composition, echogenicity, margin and calcification of the nodule were observed from ultrasound thyroid nodule images and evaluated by two physicians with more than 5 years of experience in thyroid ultrasound diagnosis according to ACR TI-RADS and Kwak TI-RADS. In case of disagreements, the physicians reached a consensus through consulting a third person.

### Composition

Cystic: cystic or almost completely cystic. Spongiform: the presence of very small cysts (50% of the nodule’s volume) that are akin to the fluidfilled spaces in a wet sponge. Solid and cystic: combines 2 features from the lexicon, regardless of the proportion of solid versus cystic components. Solid: solid or almost completely solid [16].

### Echogenicity

Hypoechoic: hypoechoic relative to thyroid parenchyma (hypoechoic relative to adjacent anterior neck muscle was also involved). Marked hypoechoic: more hypoechoic than strap muscles. Hyperechoic: hyperechoic relative to thyroid parenchyma. Anechoic: absent from the lexicon; applied to cystic or almost completely cystic nodules [17].

### Margin

Smooth or unclear: obviously discernible smooth edge. Irregular or lobulated: obviously discernible but non-smooth edge showing speculation, microlobulation (the presence of many small lobules on the surface of a nodule), or jagged appearance. Extrathyroid extension: poorly demarcated margin which cannot be obviously differentiated from adjacent thyroid tissue [17].

### Calcification

Microcalcification: calcifications that are ≤ 1 mm in diameter, with or without acoustic shadowing, brighter echo than the surrounding thyroid tissue, excluded tiny bright reflectors with a clear-cut comet-tail artifact that was considered colloid, visualized as tiny punctuate hyperechoic foci. Rim calcification: curvilinear hyperechoic structure parallel to the margin of a nodule encompassing > 120 of the circumferences. Coarse calcifications: defined as coarse hyperechoic foci > 1 mm accompanied by acoustic shadowing [17].

### Kwak-TIRADS

The number of suspicious ultrasound features calculates a score of TIRADS 3, 4A, 4B, 4C, or 5 according to the ultrasound characteristics in Kwak-TIRADS. The risk of malignancy was elevated with the increase of the number of suspicious features (solid or almost solid nodule, hypoechogenicity, irregular margins, presence of microcalcifications, and a taller than wide shape) [14]: TIRADS 3: no suspicious features (risk 1.7%); TIRADS 4A: one suspicious feature (risk 3.3%); TIRADS 4B: two suspicious features (risk 9.2%); TIRADS 4C: three or four suspicious features (risk 44.4–72.4%); TIRADS 5: five suspicious features (risk 87.5%).

### ACR TI-RADS

ACR TI-RADS includes five ultrasound features (composition, echogenicity, shape, margin, and echogenic foci) and the features are described and weighted by allocating points to get a summed score [18]: TR1: 0 points, benign (aggregate risk level 0.3%); TR2: 2 points, not suspicious (aggregate risk level 1.5%); TR3: 3 points, mildly suspicious (aggregate risk level 4.8%); TR4: 4–6 points, moderately suspicious (aggregate risk level 5.9%-12.8%); TR5: 7 points or more, highly suspicious (aggregate risk level 20.8%-68.4% for 10 points). ACR score was the total score calculated based on the scores of each features in ACR TI-RADS [18]. The detailed scores in each features were displayed as follows: Composition (0 points: Cystic or almost completely cystic; 0 points: Spongiform; 1 point: Mixed cystic and solid; 2 points: Solid or almost completely solid); Echogenicity (0 points: Anechoic; 1 point: Hyperechoic or isoechoic; 2 points: Hypoechoic; 3 points: Very hypoechoic); Shape (0 points: A/T < 1; 3 points: A/T ≥ 1); Margin (0 points: Smooth or unidentified; 2 points: Lobulated or irregular;3 points: Extra-thyroidal extension); Calcification (0 points: None or large comet-tail artifacts; 1 point: Macrocalcifications; 2 points: Peripheral (rim) calcifications; 3 points: Punctate echogenic foci).

### Statistical analysis

SAS 9.4 software was used for statistical analysis, and MedCalc software was employed to draw Receiver Operating Characteristic (ROC) curves. All statistical tests were conducted by two-side tests. Shapiro Test was applied to test the normality of the measurement data. The measurement data with normal distribution were described as Mean ± standard deviation (Mean ± SD), and the non-normal data were displayed as median and quaternary interval [M (Q_1_, Q_3_)]. The enumeration data were exhibited as n (%). χ^2^ or Fisher’s exact probability method were employed for comparisons between groups. The area under the curve (AUC) values were analyzed and the ROC curves were drawn to compare the distinguishing efficiency of ACR TI-RADS and KWAK TI-RADS on benign nodules from malignant nodules, MTC from benign nodules, PTC from benign nodules, and MTC from PTC. *P* < 0.05 was considered to be statistically significant.

## Results

### The baseline characteristics of all the participants

**Fig. 1:**
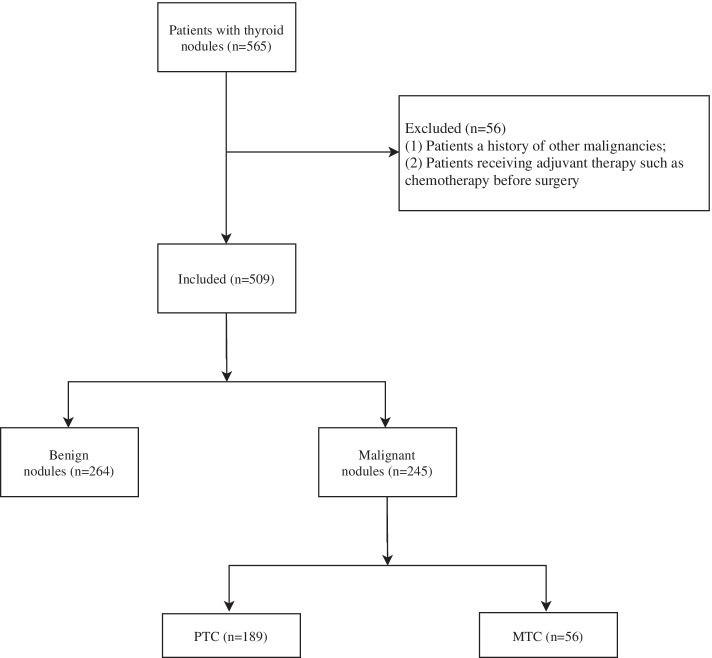
The screen process of all participants in this study

This study collected the data of 565 pathologically confirmed benign thyroid nodules, PTC and MTC patients. Among them, patients with a history of other malignancies and patients receiving adjuvant therapy such as chemotherapy before surgery were excluded (*n* = 56), and we finally involved in 509 patients. The screen process was displayed in Fig. 1.

As observed in Table 1, the average age of all participants was 48.22 years old. 264 patients were diagnosed with benign thyroid nodules, accounting for 51.87%, 245 were diagnosed with malignant thyroid nodules, accounting for 48.13%. In patients with malignant thyroid nodules,189 were PTC patients and 56 were MTC patients. The median score of ACR TI-RADS was 6 points. According to ACR TI-RADS, 54 people belonged to TR1, 30 people belonged to TR2, 66 people belonged to TR3, 116 people belonged to TR4, and 243 people belonged to TR5 class. Based on Kwak TI-RADS, 54 patients belonged to 2 category, 26 patients belonged to 3 category, and 405 patients belonged to 4 category.

**Table 1:** The baseline characteristics of all subjects

Variables	Total (*n* = 509)
Age, Mean ± SD	48.22 ± 12.31
Gender, n (%)	
Male	154 (30.26)
Female	355 (69.74)
Groups, n (%)	
Benign nodules	264 (51.87)
Malignant nodules	245 (48.13)
PTC	189 (77.14)
MTC	56 (22.86)
ACR TI-RADS score, M(Q_1_,Q_3_)	6 (3,9)
ACR TI-RADS,n(%)	
TR1	54 (10.61)
TR2	30 (5.89)
TR3	66 (12.97)
TR4	116 (22.79)
TR5	243 (47.74)
Kwak TI-RADS,n(%)	
TIRADS 2	54 (10.61)
TIRADS 3	26 (5.11)
TIRADS 4	405 (79.57)
4a	85 (20.99)
4b	80 (19.75)
4c	240 (59.26)
TIRADS 5	24 (4.72)

*ACR* American College of Radiology, *TI-RADS* Thyroid Imaging Report and Data System, *MTC* Medullary thyroid carcinoma, *PTC* Papillary thyroid carcinoma

### Comparisons of baseline data, laboratory inspection data and sonographic features in patients between benign and malignant nodules groups

As exhibited in Table 2, the mean age of patients in the malignant nodules group was lower than the benign nodules group (46.22 years vs 50.08 years). As for the examination of tumor markers, the proportions of people with positive CEA (12.65% vs 2.27%) and CT (12.24% vs 0.76%) were statistically higher in the malignant nodules group than the benign nodules group. As for the thyroid function examination, the proportions of patients with positive TPOAb (14.69% vs 4.17%), TGAb (11.02% vs 4.17%) and TRAb (84.90% vs 75.38%) was higher in the malignant nodules group than the benign nodules group. The maximum diameter of patients in the malignant nodules was smaller than the benign nodules (0.90 cm vs 3.00 cm). The distributions of patients in different composition, echogenicity, margin, calcification state group was statistically different between the malignant nodules group and the benign nodules group. The proportions of patients with shape A/T ≥ 1 (42.04% vs 8.71%) and patients with metastatic lymph nodes (23.67% vs 0%) in malignant nodules group was higher than in benign nodules group (Table 2).

**Table 2:** Comparisons of baseline data and laboratory inspection data in patients between benign and malignant nodules groups

Variable	Total (*n* = 509)	Groups		Statistical magnitude	*P*
		Benign nodule (*n* = 264)	Malignant nodule (*n* = 245)		
**Age, Mean ± SD**	48.22 ± 12.31	50.08 ± 12.32	46.22 ± 12.00	t = 3.580	< 0.001
**Gender, n (%)**				χ^2^ = 2.312	0.128
Male	154 (30.26)	72 (27.27)	82 (33.47)		
Female	355 (69.74)	192 (72.73)	163 (66.53)		
**Tumor biomarkers**					
CEA, n (%)				χ^2^ = 35.577	** < .001**
Negative	385 (75.64)	194 (73.48)	191 (77.96)		
Positive	37 (7.27)	6 (2.27)	31 (12.65)		
Unknown	87 (17.09)	64 (24.24)	23 (9.39)		
CT, n (%)				χ^2^ = 46.369	** < .001**
Negative	383 (75.25)	192 (72.73)	191 (77.96)		
Positive	32 (6.29)	2 (0.76)	30 (12.24)		
Unknown	94 (18.47)	70 (26.52)	24 (9.80)		
**Thyroid function**					
TSH, n(%)				χ^2^ = 1.979	0.372
Negative	468 (91.94)	247 (93.56)	221 (90.20)		
Positive	32 (6.29)	13 (4.92)	19 (7.76)		
Unknown	9 (1.77)	4 (1.52)	5 (2.04)		
TPOAb, n(%)				χ^2^ = 16.880	** < .001**
Negative	443 (87.03)	242 (91.67)	201 (82.04)		
Positive	47 (9.23)	11 (4.17)	36 (14.69)		
Unknown	19 (3.73)	11 (4.17)	8 (3.27)		
TGAb, n(%)				χ^2^ = 9.051	**0.011**
Negative	453 (89.00)	242 (91.67)	211 (86.12)		
Positive	38 (7.47)	11 (4.17)	27 (11.02)		
Unknown	18 (3.54)	11 (4.17)	7 (2.86)		
TRAb, n(%)				χ^2^ = 7.287	**0.026**
Negative	3 (0.59)	2 (0.76)	1 (0.41)		
Positive	407 (79.96)	199 (75.38)	208 (84.90)		
Unknown	99 (19.45)	63 (23.86)	36 (14.69)		
**Thyroid nodule**					
Maximum diameter of nodule, M (Q_1_, Q_3_)	1.80 (0.80, 3.40)	3.00 (1.70, 4.00)	0.90 (0.60, 1.70)	Z = -11.205	< 0.001
Composition, n (%)				-	< 0.001
Cystic	54 (10.61)	54 (20.45)	0 (0.00)		
Spongy	4 (0.79)	4 (1.52)	0 (0.00)		
Solid Cystic	72 (14.15)	60 (22.73)	12 (4.90)		
Solid	379 (74.46)	146 (55.30)	233 (95.10)		
Echogenicity, n (%)				χ^2^ = 155.837	< 0.001
Anechoic	55 (10.81)	55 (20.83)	0 (0.00)		
Hyperechoic	86 (16.90)	80 (30.30)	6 (2.45)		
Hypoechoic	341 (66.99)	125 (47.35)	216 (88.16)		
Markedly Hypoechoic	27 (5.30)	4 (1.52)	23 (9.39)		
Shape, n (%)				χ^2^ = 75.780	< 0.001
A/T < 1	383 (75.25)	241 (91.29)	142 (57.96)		
A/T ≥ 1	126 (24.75)	23 (8.71)	103 (42.04)		
Margin, n(%)				χ^2^ = 128.221	< 0.001
Smooth or unclear	367 (72.10)	247 (93.56)	120 (48.98)		
Irregular or lobulated	107 (21.02)	17 (6.44)	90 (36.73)		
Extrathyroid extension	35 (6.88)	0 (0.00)	35 (14.29)		
Calcification, n (%)				χ^2^ = 132.228	< 0.001
None	270 (53.05)	193 (73.11)	77 (31.43)		
Coarse calcifications	47 (9.23)	34 (12.88)	13 (5.31)		
Rim calcifications	12 (2.36)	4 (1.52)	8 (3.27)		
Micro calcifications	180 (35.36)	33 (12.50)	147 (60.00)		
**Metastatic lymph nodes, n (%)**				χ^2^ = 70.535	< 0.001
No	451 (88.61)	264 (100.00)	187 (76.33)		
Yes	58 (11.39)	0 (0.00)	58 (23.67)		

*CEA* Carcinoma embryonic antigen, *CT* Calcitonin, *TSH* Thyroid stimulating hormone, *TRAb* Thyrotropin receptor antibody, *TPOAb* Thyroid peroxidase antibody, *TGAb* Thyroglobulin antibodies

### Comparisons of baseline data, laboratory inspection data and sonographic features among MTC, PTC and benign nodules groups

According to the data in Table 3, the mean age of the MTC group was younger than the benign nodules group (48.21 years vs 50.08 years). The proportions of patients with positive CEA (48.21% vs 2.12% vs 2.27%) and CT (51.79% vs 0.53% vs 0.76%) were statistically different in the MTC group, the PTC group and the benign thyroid group. As for the thyroid function examination, the proportions of patients with positive TPOAb (12.50% vs 15.34% vs 4.17%), TGAb (7.14% vs 12.17% vs 4.17%) and TRAb (48.21% vs 95.77% vs 75.38%) were statistically different among the MTC group, the PTC group and benign nodules group. The maximum diameter of patients in the MTC group was smaller than the benign nodules (1.75 cm vs 3.00 cm). The proportions of patients with different composition, echogenicity, margin, calcification state, and shape in MTC group was statistically different from the PTC group or the benign nodules group (Table 2).

**Table 3:** Comparisons of the data in patients with benign nodules, PTC or MTC

Variable	Total (*n* = 509)	Benign nodule (*n* = 264)	PTC (*n* = 189)	MTC (*n* = 56)	Statistical magnitude	*P*
**Age, Mean ± SD**	48.22 ± 12.31	50.08 ± 12.32	45.62 ± 11.94	48.21 ± 12.09	F = 7.388	< .001
**Gender, n (%)**					χ^2^ = 4.300	0.116
Male	154 (30.26)	72 (27.27)	59 (31.22)	23 (41.07)		
Female	355 (69.74)	192 (72.73)	130 (68.78)	33 (58.93)		
**Tumor biomarkers**						
CEA, n (%)					χ^2^ = 122.238	** < .001**
Negative	385 (75.64)	194 (73.48)	172 (91.01)	19 (33.93)		
Positive	37 (7.27)	6 (2.27)	4 (2.12)	27 (48.21)		
Unknown	87 (17.09)	64 (24.24)	13 (6.88)	10 (17.86)		
CT, n (%)					χ^2^ = 152.534	** < .001**
Negative	383 (75.25)	192 (72.73)	172 (91.01)	19 (33.93)		
Positive	32 (6.29)	2 (0.76)	1 (0.53)	29 (51.79)		
Unknown	94 (18.47)	70 (26.52)	16 (8.47)	8 (14.29)		
**Thyroid function**						
TSH, n (%)					Fisher	0.151
Negative	468 (91.94)	247 (93.56)	171 (90.48)	50 (89.29)		
Positive	32 (6.29)	13 (4.92)	16 (8.47)	3 (5.36)		
Unknown	9 (1.77)	4 (1.52)	2 (1.06)	3 (5.36)		
TPOAb, n (%)					χ^2^ = 27.822	** < .001**
Negative	443 (87.03)	242 (91.67)	158 (83.60)	43 (76.79)		
Positive	47 (9.23)	11 (4.17)	29 (15.34)	7 (12.50)		
Unknown	19 (3.73)	11 (4.17)	2 (1.06)	6 (10.71)		
TGAb, n (%)					χ^2^ = 22.977	** < .001**
Negative	453 (89.00)	242 (91.67)	165 (87.30)	46 (82.14)		
Positive	38 (7.47)	11 (4.17)	23 (12.17)	4 (7.14)		
Unknown	18 (3.54)	11 (4.17)	1 (0.53)	6 (10.71)		
TRAb, n (%)					χ^2^ = 74.632	** < .001**
Negative	3 (0.59)	2 (0.76)	1 (0.53)	0 (0.00)		
Positive	407 (79.96)	199 (75.38)	181 (95.77)	27 (48.21)		
Unknown	99 (19.45)	63 (23.86)	7 (3.70)	29 (51.79)		
**Thyroid nodule**						
Maximum diameter of nodule, M (Q_1_, Q_3_)	1.80 (0.80, 3.40)	3.00 (1.70, 4.00)	0.80 (0.60, 1.40)	1.75 (0.90, 3.15)	Z = 5.137	< .001
Composition, n (%)					-	< 0.001
Cystic	54 (10.61)	54 (20.45)	0 (0.00)	0 (0.00)		
Spongy	4 (0.79)	4 (1.52)	0 (0.00)	0 (0.00)		
Solid Cystic	72 (14.15)	60 (22.73)	3 (1.59)	9 (16.07)		
Solid	379 (74.46)	146 (55.30)	186 (98.41)	47 (83.93)		
Echogenicity, n (%)					-	< 0.001
Anechoic	55 (10.81)	55 (20.83)	0 (0.00)	0 (0.00)		
Hyperechoic	86 (16.90)	80 (30.30)	4 (2.12)	2 (3.57)		
Hypoechoic	341 (66.99)	125 (47.35)	172 (91.01)	44 (78.57)		
Markedly Hypoechoic	27 (5.30)	4 (1.52)	13 (6.88)	10 (17.86)		
Shape, n (%)					χ^2^ = 22.950	< 0.001
A/T < 1	383 (75.25)	241 (91.29)	94 (49.74)	48 (85.71)		
A/T ≥ 1	126 (24.75)	23 (8.71)	95 (50.26)	8 (14.29)		
Margin, n (%)					χ^2^ = 7.555	< 0.001
Smooth or unclear	367 (72.10)	247 (93.56)	87 (46.03)	33 (58.93)		
Irregular or lobulated	107 (21.02)	17 (6.44)	78 (41.27)	12 (21.43)		
Extra thyroid extension	35 (6.88)	0 (0.00)	24 (12.70)	11 (19.64)		
Calcification, n (%)					-	< 0.001
None	270 (53.05)	193 (73.11)	59 (31.22)	18 (32.14)		
Coarse calcifications	47 (9.23)	34 (12.88)	11 (5.82)	2 (3.57)		
Rim calcifications	12 (2.36)	4 (1.52)	7 (3.70)	1 (1.79)		
Micro calcifications	180 (35.36)	33 (12.50)	59 (31.22)	18 (32.14)		
** Metastatic lymph nodes, n (%)**					χ^2^ = 5.825	< 0.001
No	451 (88.61)	264 (100.00)	151 (79.89)	36 (64.29)		
Yes	58 (11.39)	0 (0.00)	38 (20.11)	20 (35.71)		

*CEA* Carcinoma embryonic antigen, *CT* Calcitonin, *TSH* Thyroid stimulating hormone, *TRAb* Thyrotropin receptor antibody, *TPOAb* Thyroid peroxidase antibody, *TGAb* Thyroglobulin antibodies

### Discrimination values of ACR score, ACR TI-RADS and Kwak TI-RADS for malignant and benign nodules

**Fig. 2:**
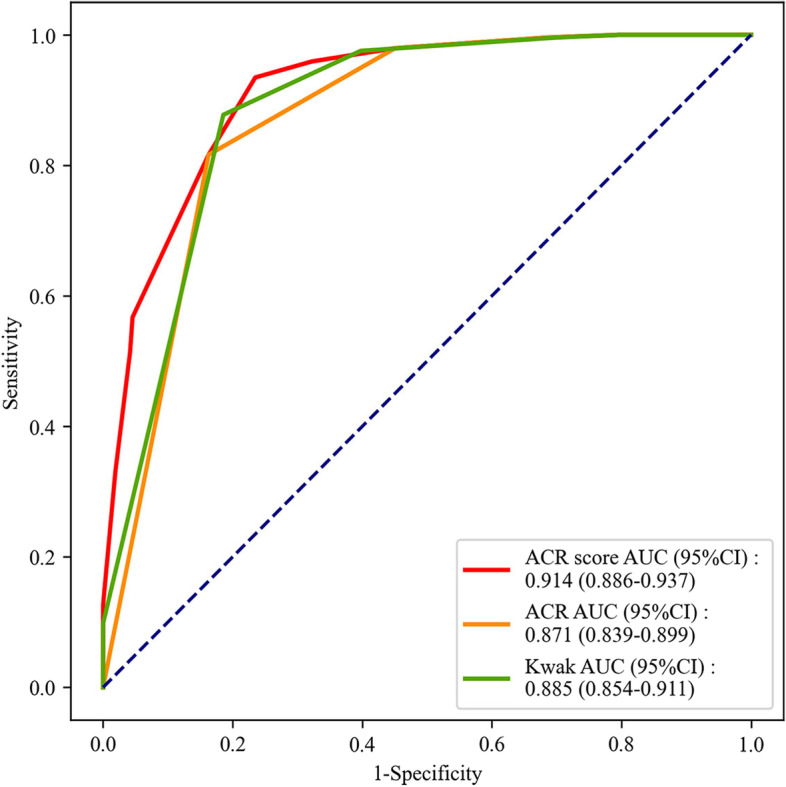
The ROC curve of the diagnostic efficiencies of ACR score, ACR TI-RADS and Kwak TI-RADS for malignant nodules in patients with benign nodules or malignant nodules

From the results in Table 4, we identified that the distributions of participants in different malignant suspicious degrees based on ACR TI-RADS or Kwak TI-RADS in the malignant nodules group were different from the benign nodules group. The AUC values of ACR score, ACR TI-RADS and Kwak TI-RADS for distinguishing malignant nodules from benign nodules were 0.914 (95%CI: 0.886–0.937), 0.871 (95%CI: 0.839–0.899) and 0.885 (95%CI: 0.854–0.911), respectively (Fig. 2). The cut-off points were > 5, > TR4 and > 4b. The sensitivities of ACR score, ACR TI-RADS and Kwak TI-RADS were 0.935 (95%CI: 0.896–0.962), 0.816 (95%CI: 0.762–0.863) and 0.878 (95%CI: 0.830–0.916), respectively. The negative predictive values (NPVs) of ACR score, ACR TI-RADS and Kwak TI-RADS were 0.927 (95%CI: 0.892–0.961), 0.831 (95%CI: 0.786–0.876), and 0.878 (95%CI: 0.837–0.919), respectively. According to the results of DeLong test, the diagnostic performance of ACR score (Z = 4.176, *P* < 0.001) for distinguishing malignant nodules from benign nodules was better than Kwak TI-RADS, but the efficacy of ACR TI-RADS for distinguishing malignant nodules from benign nodules was not better than Kwak TI-RADS (Table 5).

**Table 4:** Comparison of pathological data of ACR, ACR TI-RADS and Kwak TI-RADS on benign nodules and malignant nodules

Variable	Total (*n* = 509)	Groups		Statistical magnitude	*P*
		Benign nodule (*n* = 264)	Malignant nodule (*n* = 245)		
ACR score, M (Q_1_, Q_3_)	6 (3, 9)	3 (2, 5)	9 (7, 10)	Z = -16.248	< 0.001
ACR TI-RADS, n (%)				Z = -15.471	< 0.001
TR1	54 (10.61)	54 (20.45)	0 (0.00)		
TR2	30 (5.89)	29 (10.98)	1 (0.41)		
TR3	66 (12.97)	62 (23.48)	4 (1.63)		
TR4	116 (22.79)	76 (28.79)	40 (16.33)		
TR5	243 (47.74)	43 (16.29)	200 (81.63)		
Kwak TI-RADS, n (%)				Z = -15.956	< 0.001
TIRADS 2	54 (10.61)	54 (20.45)	0 (0.00)		
TIRADS 3	26 (5.11)	25 (9.47)	1 (0.41)		
TIRADS 4a	85 (16.70)	80 (30.30)	5 (2.04)		
TIRADS 4b	80 (15.72)	56 (21.21)	24 (9.80)		
TIRADS 4c	240 (47.15)	49 (18.56)	191 (77.96)		
TIRADS 5	24 (4.72)	0 (0.00)	24 (9.80)

**Table 5:** Diagnostic performances of ACR score, ACR TI-RADS and Kwak TI-RADS in the diagnosis of benign and malignant nodules

Tools	AUC area(95%CI)	Cutoff	Sensitivity (95%CI)	Specificity (95%CI)	PPV (95%CI)	NPV (95%CI)	Z	P
ACR score	0.914 (0.886–0.937)	> 5	0.935 (0.896–0.962)	0.765 (0.709–0.815)	0.787 (0.740–0.834)	0.927 (0.892–0.961)	4.176	< 0.001
ACR TI-RADS	0.871 (0.839–0.899)	> TR4	0.816 (0.762–0.863)	0.837 (0.787–0.880)	0.823 (0.775–0.871)	0.831 (0.786–0.876)	2.319	0.020
Kwak TI-RADS	0.885 (0.854–0.911)	> 4b	0.878 (0.830–0.916)	0.814 (0.762–0.859)	0.814 (0.767–0.861)	0.878 (0.837–0.919)		

*ACR* American College of Radiology, *TI-RADS* Thyroid Imaging Report and Data System, *PPV* Positive predictive value, *NPV* Negative predictive value

### Discrimination values of ACR score, ACR TI-RADS and Kwak TI-RADS for PTC or MTC and patients with benign nodules

**Fig. 3:**
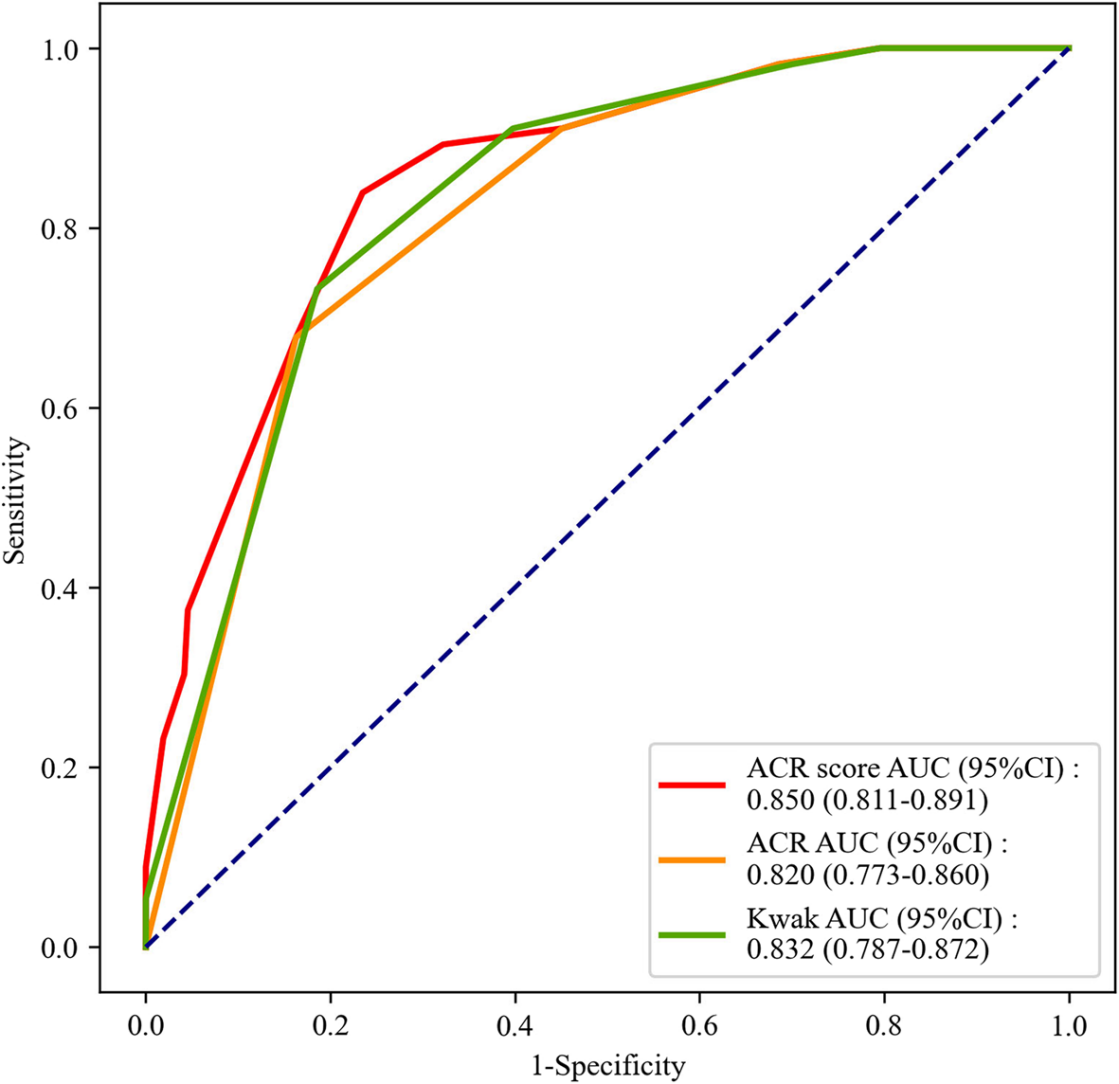
The ROC curve of the efficiencies of ACR score, ACR TI-RADS and Kwak TI-RADS in distinguishing MTC from benign nodules

**Fig. 4:**
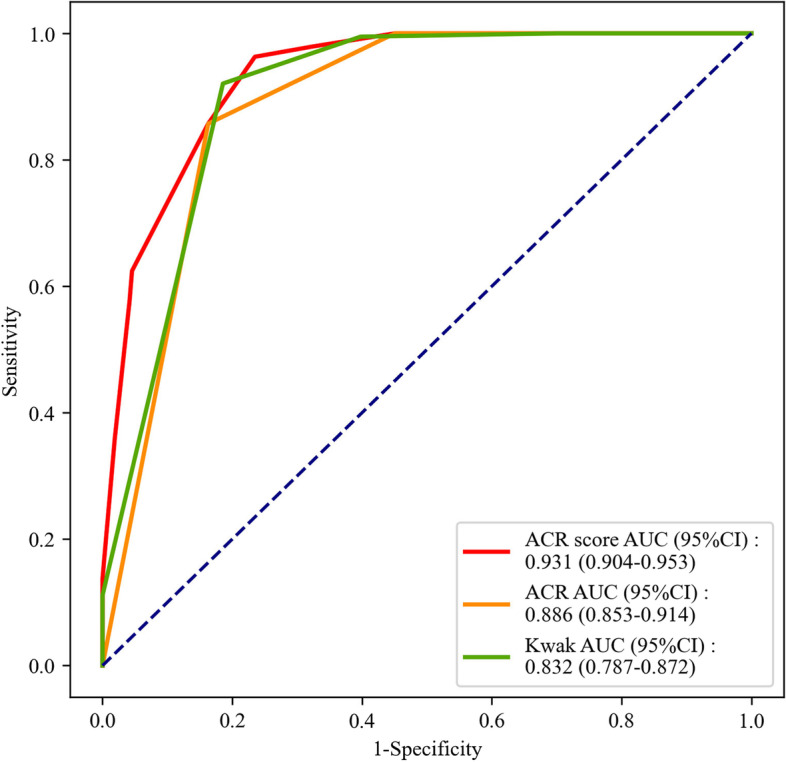
The ROC curve of the efficiencies of ACR score, ACR TI-RADS and Kwak TI-RADS in distinguishing PTC from benign nodules

The distributions of patients with benign nodules, PTC or MTC based on ACR score, ACR TI-RADS and Kwak TI-RADS were shown in Table 6. In the benign nodules and MTC populations, the AUC values of ACR score, ACR TI-RADS and Kwak TI-RADS for distinguishing MTC from benign nodules were 0.850 (95%CI: 0.811–0.891), 0.820 (95%CI: 0.773–0.860) and 0.832 (95%CI: 0.787–0.872), respectively (Fig. 3). The cut-off points were > 5, > TR4 and > 4b. According to the Delong test, there was no statistical difference in the efficiency for differentiating patients with MTC from patients with benign nodules between ACR TI-RADS and Kwak TI-RADS, while ACR score was better than Kwak TI-RADS in the differentiation of patients with MTC from patients with benign nodules (Z = 2.404, *P* = 0.016) (Table 7). In benign nodules and PTC populations, the AUC values of ACR score, ACR TI-RADS and Kwak TI-RADS for differentiating PTC from patients with benign nodules were 0.931 (95%CI: 0.904–0.953), 0.886 (95%CI: 0.853–0.914) and 0.900 (95%CI:0.876–0.925), respectively (Fig. 4), and the cutoff point was > 5, > TR4 and > 4b, respectively. The sensitivities were 0.963 (95%CI: 0.925–0.985), 0.857 (95%CI: 0.799–0.904) and 0.921 (95%CI: 0.882–0.959), respectively. The NPVs were 0.967 (95%CI: 0.942–0.991), 0.891 (95%CI: 0.852–0.930), and 0.935 (95%CI: 0.903–0.967), respectively. The results of Delong Test showed that the discrimination efficiency of ACR score (Z = 2.869, *P* = 0.004) and ACR TI-RADS (Z = 2.235, *P* = 0.025) for distinguishing PTC was better than for distinguishing MTC from benign nodules (Table 7).

**Table 6:** Comparison of pathological data of ACR, ACR TI-RADS and Kwak TI-RADS on benign nodules, PTC and MTC

Variables	Total (*n* = 509)	Groups		
		Benign nodule (*n* = 264)	PTC (*n* = 189)	MTC (*n* = 56)
ACR score, M(Q_1_,Q_3_)	6 (3,9)	3 (2,5)	9 (7,10)	7 (6,9)
ACR TI-RADS, n (%)				
TR1	54 (10.61)	54 (20.45)	0 (0.00)	0 (0.00)
TR2	30 (5.89)	29 (10.98)	0 (0.00)	1 (1.79)
TR3	66 (12.97)	62 (23.48)	0 (0.00)	4 (7.14)
TR4	116 (22.79)	76 (28.79)	27 (14.29)	13 (23.21)
TR5	243 (47.74)	43 (16.29)	162 (85.71)	38 (67.86)
Kwak TI-RADS, n(%)				
TI-RADS 2	54 (10.61)	54 (20.45)	0 (0.00)	0 (0.00)
TI-RADS 3	26 (5.11)	25 (9.47)	0 (0.00)	1 (1.79)
TI-RADS 4a	85 (16.70)	80 (30.30)	1 (0.53)	4 (7.14)
TI-RADS 4b	80 (15.72)	56 (21.21)	14 (7.41)	10 (17.86)
TI-RADS 4c	240 (47.15)	49 (18.56)	153 (80.95)	38 (67.86)
TI-RADS 5	24 (4.72)	0 (0.00)	21 (11.11)	3 (5.36)

*ACR* American College of Radiology, *TI-RADS* Thyroid Imaging Report and Data System, *MTC* Medullary thyroid carcinoma, *PTC* Papillary thyroid carcinoma

**Table 7:** Diagnostic performances of ACR score, ACR TI-RADS and Kwak TI-RADS in the diagnosis of MTC or PTC

Variables	AUC (95%CI)	Cutoff	Sensitivity (95%CI)	Specificity (95%CI)	PPV (95%CI)	NPV (95%CI)	Z	*P*	Z^*^	P^*^
ACR score^a^	0.850 (0.811–0.891)	> 5	0.839 (0.717–0.924)	0.765 (0.709–0.815)	0.431 (0.709–0.815)	0.957 (0.709–0.815)	2.404	0.016		
ACR TI-RADS^a^	0.820 (0.773–0.860)	> TR4	0.679 (0.540–0.797)	0.837 (0.787–0.880)	0.469 (0.360–0.578)	0.925 (0.891–0.958)	1.497	0.134		
Kwak TI-RADS^a^	0.832 (0.787–0.872)	> 4b	0.732 (0.597–0.842)	0.814 (0.762–0.859)	0.456 (0.353–0.558)	0.935 (0.903–0.967)	-	-		
ACR score^b^	0.931 (0.904–0.953)	> 5	0.963 (0.925–0.985)	0.765 (0.709–0.815)	0.746(0.691–0.801)	0.967(0.942–0.991)	1.754	0.079	2.869	0.004
ACR TI-RADS^b^	0.886 (0.853–0.914)	> TR4	0.857 (0.799–0.904)	0.837 (0.787–0.880)	0.790(0.735–0.846)	0.891(0.852–0.930)	0.701	0.483	2.235	0.025
Kwak TI-RADS^b^	0.900 (0.876–0.925)	> 4b	0.921 (0.882–0.959)	0.814 (0.767–0.861)	0.780(0.726–0.835)	0.935(0.903–0.967)	-	-		

Z and *P* depicted the results compared with Kwak TI-RADS

Z^*^ and P^*^ revealed the results of ACR score ^b^ and ACR TI-RADS ^b^ compared with ACR score ^a^ and ACR TI-RADS ^a^

*ACR* American College of Radiology, *TI-RADS* Thyroid Imaging Report and Data System, *PPV* Positive predictive value, *NPV* Negative predictive value, *MTC* Medullary thyroid carcinoma, *PTC* Papillary thyroid carcinoma

^a^Diagnostic performance of ACR score, ACR TI-RADS and Kwak TI-RADS for MTC compared with benign nodules

^b^Diagnostic performance of ACR score, ACR TI-RADS and Kwak TI-RADS for PTC compared with benign nodules

### Discrimination values of ACR score, ACR TI-RADS and Kwak TI-RADS for MTC and PTC

**Fig. 5:**
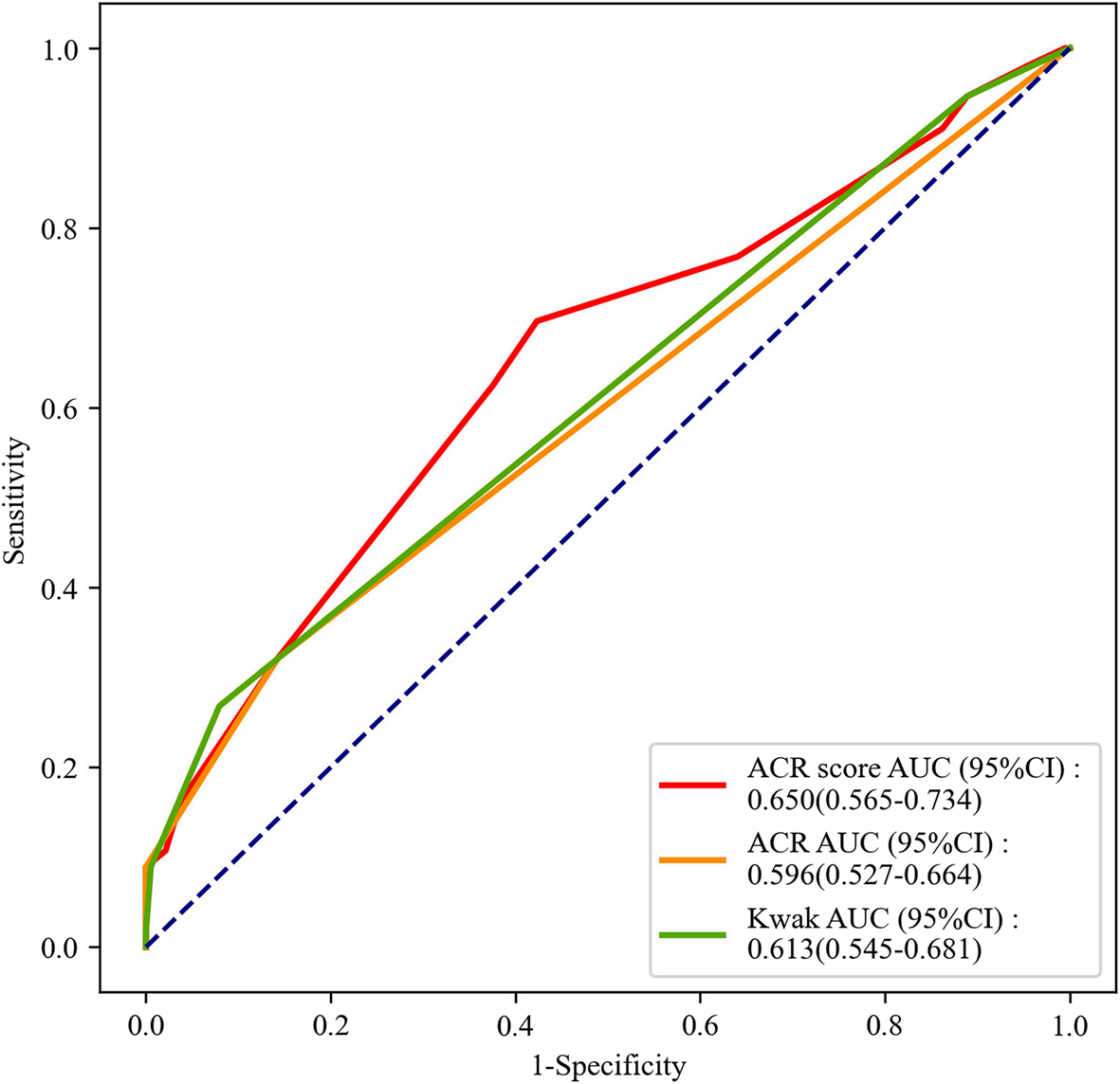
The ROC curve of the diagnostic efficiencies of ACR score, ACR TI-RADS and Kwak TI-RADS for MTC patients with MTC or PTC

In patients with MTC or PTC, the AUC values of ACR score, ACR TI-RADS and Kwak TI-RADS for distinguishing patients with MTC from patients with PTC were 0.650 (95%CI: 0.565–0.734), 0.596 (95%CI: 0.527–0.664), and 0.613 (95%CI: 0.545–0.681), respectively (Fig. 5). The NPVs were 0.865 (95%CI: 0.805–0.925), 0.810 (95%CI: 0.756–0.864), and 0.809 (95%CI: 0.757–0.862), respectively. All the false negatives for malignant nodules of ACR TI-RADS were MTC and none PTC. The cutoff points were ≤ 8, ≤ TR4 and ≤ 4b, respectively. The results from Delong test revealed that there was no significant difference in ACR score (Z = 0.669, *P* = 0.504) and ACR TI-RADS (Z = 0.345, *P* = 0.730) for differentiating MTC from PTC compared with Kwak TI-RADS (Table 8).

**Table 8:** Diagnostic performances of ACR score, ACR TI-RADS and Kwak TI-RADS in the diagnosis of PTC from MTC

Variables	AUC area (95%CI)	Cutoff	Sensitivity (95%CI)	Specificity (95%CI)	PPV (95%CI)	NPV (95%CI)	Z	*P*
ACR score	0.650 (0.565–0.734)	≤ 8	0.696 (0.576–0.817)	0.577 (0.506–0.647)	0.328 (0.243–0.412)	0.865 (0.805–0.925)	0.669	0.504
ACR TI-RADS	0.596 (0.527–0.664)	≤ TR4	0.321 (0.199–0.444)	0.857 (0.807–0.907)	0.400 (0.257–0.543)	0.810 (0.756–0.864)	0.345	0.730
Kwak TI-RADS	0.613 (0.545–0.681)	≤ 4b	0.268 (0.152–0.384)	0.921 (0.882–0.959)	0.500 (0.321–0.679)	0.809 (0.757–0.862)		

*ACR* American College of Radiology, *TI-RADS* Thyroid Imaging Report and Data System, *PPV* Positive predictive value, *NPV* Negative predictive value, *MTC* Medullary thyroid carcinoma, *PTC* Papillary thyroid carcinoma

### Discrimination values of ACR score, ACR TI-RADS and Kwak TI-RADS for MTC, PTC or benign nodules

According to the data in Supplementary Table 1, the AUC values of ACR score, ACR TI-RADS and Kwak TI-RADS for the discrimination of patients with MTC, PTC or benign nodules from patients without MTC, PTC or benign nodules were 0.899 (95%CI: 0.882–0.915), 0.865 (95%CI: 0.846–0.885), and 0.873 (95%CI: 0.854–0.893), respectively (Supplementary Fig. 1). The NPVs were 0.915 (95%CI: 0.896–0.933), 0.876 (95%CI: 0.856–0.896), and 0.882 (95%CI: 0.862–0.902), respectively. As observed in the results from Delong test, ACR score had better efficiency in discriminating patients with MTC, PTC or benign nodules from patients without MTC, PTC or benign nodules than Kwak TI-RADS (Z = 1.995, *P* = 0.046), but ACR TI-RADS showed no significant difference in discriminating patients with MTC, PTC or benign nodules compared with Kwak TI-RADS (Z = 0.569, *P* = 0.570).

## Discussion

We collected the data of 509 patients diagnosed with PTC, MTC or benign thyroid nodules to evaluate the diagnostic performance of ACR score and ACR TI-RADS in different kinds of thyroid nodules especially in MTC. The results delineated that the diagnostic performances of ACR score was better than Kwak TI-RADS in distinguishing malignant nodules from benign nodules. ACR score was better than Kwak TI-RADS in diagnosis of MTC from benign nodules. ACR TI-RADS had similar diagnostic efficacy with Kwak TI-RADS in discriminating MTC or PTC from benign nodules.

Currently, the diagnosis of benign or malignant thyroid nodules in patients remains to be a challenge [19]. The TI-RADS for risk stratification is widely used for evaluating the suspicious degrees of malignant thyroid nodules through scoring the number or the combination of a variety of suspicious ultrasound criteria with high accuracy and repeatability [15, 18, 20, 21]. Globally, several standardized reporting systems have been proposed including 2015 ATA guideline, Korean Thyroid Association/Korean Society of Thyroid Radiology (KTA/KSThR) guideline, Kwak TI-RADS and ACR TI-RADS [18, 22, 23]. A previous study was conducted to compare the diagnostic ability of the KSThR guideline, European Thyroid Society (ETA) and ACR TI-RADS in distinguishing benign from malignant thyroid lesions, which revealed that KSTHR TI-RADS had the best diagnostic specificity, while ACR TI-RADS had the best sensitivity [24]. As shown in the study of Zhang et al., the AUC value of the diagnostic performance of ACR TI-RADS in thyroid nodules was 0.907, which was higher than Kwak TI-RADS (0.904), ATA guidelines (0.894) and KTA/KSThR guidelines (0.888) [25]. The sensitivity (85.7%) and NPV (98.3%) of ACR TI-RADS were both higher than EU TI-RADS and the specificity of ACR TI-RADS (51.1%) was higher than Kwak TI-RADS [26]. In this study, we found that compared with Kwak TI-RADS, ACR TI-RADS had better specificity and PPV in differentiating the benign and malignant thyroid nodules.

A recent study delineated that Kwak TI-RADS and ATA TI-RADS had similar performance in the diagnosis of MTC, but the performance was worse than in the diagnosis of PTC [13]. In the current study, the diagnosis performance of ACR TI-RADS in distinguishing MTC and PTC from benign nodules was compared with Kwak TI-RADS, which showed that the diagnosis value of ACR TI-RADS on MTC or PTC was comparable with Kwak TI-RADS, and ACR TI-RADS had better diagnosis efficiency on PTC than MTC. MTC and PTC may have some overlapping sonographic features of malignancy which may result in the misdiagnosis of MTC [27]. Here in our study, both ACR TI-RADS and Kwak TI-RADS had average diagnostic abilities in differentiating MTC from PTC. This maybe because due to the high prevalence of PTC, most of the present classification systems mainly focus on differentiating PTC [28]. In the present study, the ACR score showed better diagnostic abilities on distinguishing malignant nodules from benign nodules, MTC from benign nodules, and MTC from PTC. Kwak TI-RADS diagnosed the malignancy via calculating the malignant features, which has high sensitivity and it is simple and easy to conduct. But the malignant degree of the sonographic features is not shown, so it was more applied for screening different kinds of thyroid nodules [29]. ACR TI-RADS was calculated via cumulative scores of different sonographic features, different features were scored differently, reflecting the malignant risk degrees of different sonographic features, which is more complicated and had higher specificity, it is more commonly used for the diagnosis of different kinds of thyroid nodules [29].

The present study assessed the discriminative efficacies of ACR score and ACR TI-RADS on malignant nodules from benign nodules, MTC from PTC or benign nodules, as well as PTC from benign nodules compared with Kwak TI-RADS. ACR score used single score to diagnose malignant nodules from benign nodules, and MTC from benign nodules with AUC values of 0.914 and 0.850, respectively, which was better than ACR TI-RADS and Kwak TI-RADS. The AUC value of ACR score were 0.650 in differentiating MTC from PTC, and in the future, the application of ACR score combined with other diagnostic methods might improve the diagnostic performance. ACR TI-RADS had a higher diagnostic value for benign nodules and malignant nodules than Kwak TI-RADS. MTC is a highly malignant, and patients underwent surgery can be cured except the advanced MTC, so early diagnosis is important for improving the prognosis [30]. The findings of this study might provide a reference for using ACR TI-RADS for newly diagnosed thyroid nodule patients to distinguish malignant nodules, benign nodules, PTC and MTC based on ultrasound data. For patients with highly-suspected MTC based on the results from ACR TI-RADS, CT assay was recommended as it is considered to be a sensitive and specific marker for the diagnosis of MTC [31]. Using of ACR TI-RADS might also help reduce the unnecessary biopsies in FNA, and decrease the waste of medical resources and the psychological burden to patients.

Several limitations existed in this study. Firstly, the sample size was small, which might decrease the statistical power of our results. Secondly, ACR score was derived from ACR TI-RADS, the efficacy of it should be verified. Studies with large scale of sample size were required to validate the results of our study.

## Conclusions

Our study collected the data of 509 patients diagnosed with PTC, MTC or benign thyroid nodules and assessed the diagnostic performances of ACR score and ACR TI-RADS. The data depicted that the ACR score performed the best, followed ex aequo by the ACR and Kwak TI-RADS in discriminating patients with malignant nodules from benign nodules and patients with MTC from PTC. The findings of our study might provide a reference for the application of ACR score and ACR TI-RADS in the diagnosis of MTC, PTC and benign nodules.

## Supplementary Information

**Additional file 1.**

**Additional file 2.**

## Data Availability

The datasets generated and/or analysed during the current study are not publicly available due to limitations of ethical approval involving the patient data and anonymity but are available from the corresponding author on reasonable request.
