# Supplementary material for: Clinical diagnostic value of American College of Radiology thyroid imaging report and data system in different kinds of thyroid nodules
Source: BMC Endocr Disord. 2022 May 31;22:145. doi: 10.1186/s12902-022-01053-z (PMC9158315; doi:10.1186/s12902-022-01053-z)
Supplement: Supplementary file 1 — Additional file 1. [file 12902_2022_1053_MOESM1_ESM.docx]

Supplementary Table 1 Comparisons of diagnostic performances of ACR score, ACR TI-RADS and Kwak TI-RADS in the diagnosis of benign nodules, PTC and MTC

| Variables | AUC (95%CI) | Sensitivity (95%CI) | Specificity (95%CI) | PPV (95%CI) | NPV (95%CI) | Z | *P* |
| --- | --- | --- | --- | --- | --- | --- | --- |
| ACR score | 0.899(0.882-0.915) | 0.853 (0.822-0.883) | 0.789 (0.764-0.814) | 0.669 (0.633-0.705) | 0.915 (0.896-0.933) | 1.995 | 0.046 |
| ACR TI-RADS | 0.865(0.846-0.885) | 0.752 (0.715-0.790) | 0.876 (0.856-0.896) | 0.752 (0.715-0.790) | 0.876 (0.856-0.896) | 0.569 | 0.570 |
| Kwak TI-RADS | 0.873(0.854-0.893) | 0.764 (0.727-0.801) | 0.882 (0.862-0.902) | 0.764 (0.727-0.801) | 0.882 (0.862-0.902) |  |  |

Compared with Kwak TI-RADS

ACR: American College of Radiology, TI-RADS: Thyroid Imaging Report and Data System, PPV: positive predictive value, NPV: negative predictive value, MTC: medullary thyroid carcinoma, PTC: papillary thyroid carcinoma
