# Supplementary material for: Clinical diagnostic value of American College of Radiology thyroid imaging report and data system in different kinds of thyroid nodules
Source: BMC Endocr Disord. 2022 May 31;22:145. doi: 10.1186/s12902-022-01053-z (PMC9158315; doi:10.1186/s12902-022-01053-z)
Supplement: Supplementary file 2 — Additional file 2. [file 12902_2022_1053_MOESM2_ESM.pdf]

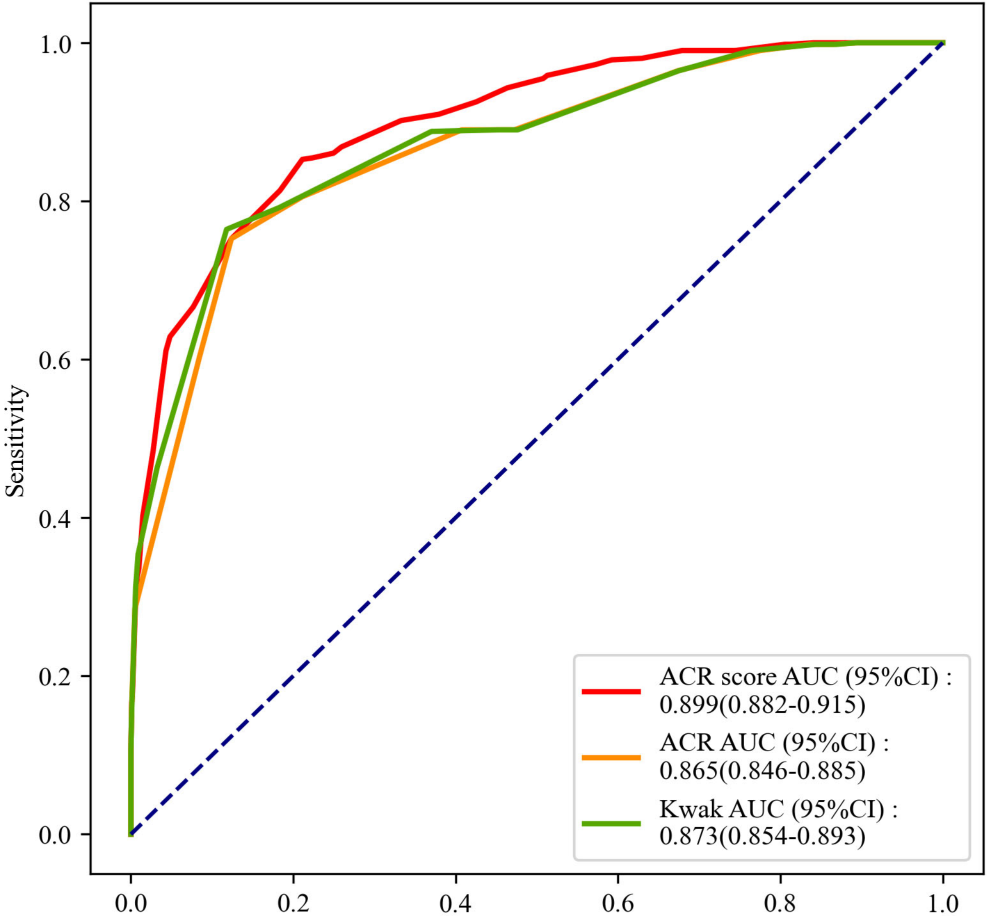

Supplementary Figure 1

The ROC curve of the discriminative efficiencies of ACR score, ACR TI-RADS and Kwak TI-RADS in benign nodules, PTC or MTC from patients without benign nodules, PTC or MTC.
